# Genome-Wide Bioinformatics Identification and Functional Analysis of the *3-ketoacyl-CoA Synthase* (*KCS*) Gene Family in *Rosa* × *hybrida*, with Focus on *RcKCS6*

**DOI:** 10.3390/genes17030286

**Published:** 2026-02-27

**Authors:** Yiwei Peng, Jianling Lv, Jiamei Zou, Jing Meng, Xuejiao Li, Jingli Zhang, Gengyun Li, Yongfu Peng, Liang Wei, Bin Liu, Shuilian He

**Affiliations:** 1 College of Landscape and Horticulture, Yunnan Agricultural University, Kunming 650201, China; pengyiwei1106@163.com (Y.P.); lyu02148349@163.com (J.L.); 18388568786@163.com (J.Z.); mengjing2514@163.com (J.M.); lixuejiao@ynau.edu.cn (X.L.); jl200812@yeah.net (J.Z.); gengyun@fudan.edu.cn (G.L.); 2 Yunnan Aibida Horticultural Technology Co., Ltd., Kunming 650600, China; pyf@aibida.cn (Y.P.); weiliang@aibida.cn (L.W.)

**Keywords:** *3-ketoacyl-CoA synthase* (*KCS*), gene family, *Rosa* × *hybrida*, *Botrytis cinerea*, functional

## Abstract

Background/Objectives: The 3-ketoacyl-CoA synthase (KCS) enzyme is a key and rate-limiting component in the biosynthesis of very long-chain fatty acids (VLCFAs). Through controlling VLCFA production, KCS plays an essential role in plant cuticle formation. The necrotrophic fungus *Botrytis cinerea* can infect all aboveground parts of rose plants (flowers, leaves, and stems), causing severe economic losses. KCS restricts pathogen invasion by influencing cuticle formation and enhances tolerance to environmental stresses. While the *KCS* gene family has been well-studied in some plants, it remains unexplored in rose (*Rosa* × *hybrida* Hort.), a species of significant ornamental and economic value. Methods: In this study, we conducted a genome-wide analysis of the *RcKCS* gene family in rose, identifying 18 non-redundant genes. Phylogenetic, structural, and synteny analyses were performed to investigate the evolutionary relationships, gene architecture, and duplication events. The expression patterns of *RcKCS* genes in rose petals during *B. cinerea* infection were examined, and transient overexpression and silencing of *RcKCS6* were used to study its function. Results: *RcKCS6* was found to be upregulated during gray mold infection, and transient overexpression reduced lesion size on infected petals. Conclusions: Our study provides the first comprehensive analysis of the *RcKCS* gene family in rose and highlights *RcKCS6* as a potential candidate for improving resistance to gray mold in rose through molecular breeding.

## 1. Introduction

Rose (*Rosa* × *hybrida* Hort.), belonging to the Rosaceae family, is revered worldwide as the ‘Queen of Flowers’ due to its vast diversity, captivating beauty, and prolonged flowering period [1]. It is one of the most commercially significant cut flowers globally, generating billions of dollars in annual revenue and accounting for approximately 30% of the global cut flower market [2]. However, rose production faces severe challenges from various diseases, among which gray mold, caused by the necrotrophic fungus *Botrytis cinerea*, is particularly devastating. This pathogen can infect all aerial parts of the plant (flowers, leaves, and stems) and is notorious for its ability to thrive in post-harvest conditions of low temperature and high humidity, leading to substantial economic losses [3,4]. *B. cinerea* has a broad host range, capable of infecting over 235 plant species [5], and understanding the molecular basis of rose–*B. cinerea* interactions is crucial for developing effective resistance strategies. The recent availability of high-quality rose genome sequences and transcriptome datasets has facilitated the genome-wide identification and functional prediction of various gene families involved in stress responses. For example, members of the *bZIP* (e.g., *RcbZIP17*, *FabZIP46*), WRKY (e.g., *RhWRKY30*, *RcWRKY41*), and *AP2*/*ERF* (e.g., *RcERF099*) families have been implicated in gray mold resistance [6]. Preliminary functional validation has also been conducted for other families, such as *TBL* (*RcTBL16*) and *bHLH* (*RcbHLH112*). While the role of *KCS* transcription factors in stress responses has been well-documented in model plants like Arabidopsis, a comprehensive analysis of the *KCS* gene family in roses is still lacking.

Plants, as sessile organisms, have evolved sophisticated morphological and molecular mechanisms to survive in environments replete with biotic and abiotic stresses. Biotic stresses, such as pathogen infections, and abiotic stresses, including drought, salinity, and extreme temperatures, pose significant threats to plant growth, development, and productivity [7]. The plant cuticle, a hydrophobic layer covering the aerial surfaces, serves as the first line of defense against these environmental challenges [8,9]. This protective barrier, primarily composed of cutin and epicuticular waxes, plays a pivotal role in limiting non-stomatal water loss, reflecting excess ultraviolet radiation, and preventing the entry of pathogenic fungi and bacteria [10]. The structural complexity of the cuticular wax layer is derived from very long-chain fatty acids (*VLCFAs*) and their various derivatives, including alkanes, aldehydes, primary and secondary alcohols, ketones, and esters [11]. VLCFAs are synthesized in the endoplasmic reticulum by the fatty acid elongase (FAE) complex through sequential two-carbon elongation involving condensation, reduction, dehydration, and a second reduction [12]. The initial condensation reaction, which is the rate-limiting step, is catalyzed by 3-ketoacyl-CoA synthase (KCS). The resulting elongated 3-ketoacyl-CoA is then processed by the other three enzymes—ketoacyl-CoA reductase (KCR), hydroxyacyl-CoA dehydratase (HCD), and enoyl-CoA reductase (ECR)—to form a saturated *VLCFA* [13].

Since the pioneering discovery and functional characterization of *FAE1/KCS18* in *Arabidopsis thaliana*, which is essential for the synthesis of seed-specific *VLCFAs* [14], numerous *KCS* gene family members have been identified across the plant kingdom [12,13,15,16,17]. Based on amino acid sequence homology and functional characteristics, the plant *KCS* family can be phylogenetically divided into several subfamilies, such as the FAE1-like, FDH-like, and CER6-like clades [18]. Structurally, KCS proteins typically contain two conserved domains: The N-terminal FAE1/type III polyketide synthase-like domain (FAE1_CUT1_RppA, PF08541) and the C-terminal 3-hydroxyacyl-CoA dehydrogenase type III domain (ACP_syn_III_C, PF08392) [19]. The functional roles of *KCS* genes extend beyond cuticular wax biosynthesis to include the formation of suberin in roots and seed coats, the production of sphingolipids for membrane integrity, and the synthesis of storage lipids in seeds [20]. For instance, *AtKCS1* is involved in the synthesis of VLCFAs for seed triglycerides, root lipids, and sphingolipids [21], while *AtKCS2/DAISY* and *AtKCS20* have been demonstrated to function redundantly in cuticular wax and root suberin biosynthesis [22]. Similarly, *AtKCS9* and *AtKCS16* are critical for the production of wax precursors, suberin monomers, and sphingolipid bases [23,24]. Orthologs of these genes in crops also play conserved roles; for example, *CsKCS6* in citrus [25], *OsKCS6* in rice [26], and *HaKCS* in sunflower [27] are all implicated in cuticular wax deposition. Accumulating evidence underscores the importance of *KCS* genes in plant adaptive responses to environmental stresses. In Arabidopsis, *AtKCS2* and *AtKCS20* are up-regulated under osmotic stress [22]. Overexpression of *MdKCS2* in apple enhances wax accumulation and drought tolerance [28], while ectopic expression of *VvKCS* from grape in Arabidopsis improves salt and drought resilience [29,30]. In barley, silencing of *HvKCS6 seems to affect* wax composition and plant performance under water-limited conditions [31], and overexpression of *HvKCS1* increases leaf wax content and confers resistance to powdery mildew [32]. These studies collectively highlight the dual role of *KCS* enzymes in both developmental processes and stress adaptation.

This study aims to fill this knowledge gap by performing a genome-wide identification and systematic characterization of the *KCS* gene family in *Rosa × hybrida.* We analyzed their chromosomal locations, phylogenetic relationships, gene structures, conserved motifs, gene duplication events, and promoter cis-elements. Furthermore, we investigated the expression profiles of *RcKCS* genes in response to *B. cinerea* infection in the *Rosa × hybrida* cultivar ‘Jumilia’.

## 2. Materials and Methods

### 2.1. Plant Materials

Petals of *Rosa × hybrida* cultivar ‘Jumilia’ were used in this study. Fresh commercial cut flowers were purchased from the Dounan Flower Market (Kunming, Yunnan Province, China). Upon arrival at the laboratory, petals were immediately excised from fully opened flowers at a uniform developmental stage and used for subsequent experimental treatments, including *B. cinerea* inoculation, transient overexpression, and virus-induced gene silencing (VIGS) assays. Only healthy petals without visible mechanical damage or disease symptoms were selected to ensure experimental consistency and reproducibility. All experiments were conducted using freshly collected petals under controlled laboratory conditions.

### 2.2. Identification of KCS Genes in the Rose Genome

To comprehensively identify all potential *KCS* genes in the rose genome, a dual approach was employed. First, the hidden Markov model (HMM) profiles for the two conserved *KCS* domains, FAE1_CUT1_RppA (PF08541) and ACP_syn_III_C (PF08392), were retrieved from the Pfam database (https://pfam.xfam.org). These HMM profiles were used to search against the proteome of *rose* using HMMER software (version 3.3.2; http://hmmer.org/download.html) with a stringent E-value cutoff of 1 × 10^−5^ [13,33]. Second, a local BLASTp (version 2.14.1) search was performed using all known *KCS* protein sequences from *A. thaliana* as queries against the rose proteome, with an E-value threshold set at 1 × 10^−10^ [17]. KCS protein sequences of *A. thaliana* were downloaded from the Arabidopsis genome database TAIR (https://www.arabidopsis.org/). The results from both approaches were combined, and redundant sequences were removed. To further verify the reliability of the candidate genes, their protein sequences were subjected to domain analysis using the SMART (Simple Modular Architecture Research Tool; http://smart.embl-heidelberg.de/), CDD (Conserved Domain Database) of NCBI (https://www.ncbi.nlm.nih.gov/Structure/cdd/wrpsb.cgi, accessed on 12 January 2025), and the Pfam database (https://pfam.xfam.org/). Only sequences that contained both characteristic KCS domains were retained for subsequent analysis.

### 2.3. Sequence Characterization, Subcellular Localization Prediction, and Phylogenetic and Evolutionary Analysis

The physicochemical properties of the identified RcKCS proteins, including the number of amino acids, molecular weight (kDa), theoretical isoelectric point (pI), instability index, and aliphatic index, were computed using the ProtParam tool available on the ExPASy server. The subcellular localization of each RcKCS protein was predicted using the WolfPSORT online tool, which analyzes amino acid sequences for sorting signals and composition. To elucidate the evolutionary relationships among KCS proteins, full-length amino acid sequences of *KCS* genes from *rose* (18), *A. thaliana* (21), and *Glycine max* (31) were retrieved (Table S1). Multiple sequence alignment was performed using the ClustalW program with default parameters. A phylogenetic tree was constructed using the Neighbor-Joining (NJ) method in MEGA software (version 11.0). The reliability of the tree topology was assessed by bootstrap analysis with 1000 replicates [17]. The final tree was visualized and annotated using the iTOL (https://itol.embl.de/upload.cgi, accessed on 12 January 2025) online tool.

### 2.4. Chromosomal Location and Collinearity Analysis

The chromosomal location information for each *RcKCS* gene, including start and end positions, was extracted from the genome annotation file (GFF3 format). The genes were renamed sequentially from *RcKCS1* to *RcKCS18* according to their physical positions on the chromosomes. The MapGene2Chromosome (MG2C) online tool was used to visualize the distribution of *RcKCS* genes on the rose chromosomes. To investigate gene duplication events, the One-Step MCScanX algorithm implemented in TBtools software (version2.400) was used to analyze the rose genome. The Advanced Circos function in TBtools was employed to visualize segmental and tandem duplication events within the rose genome. Furthermore, to explore the syntenic relationships between rose and *Arabidopsis*, a comparative genomic analysis was performed using the One-Step MCScanX function, and the results were visualized with the Dual Systeny Plot tool in TBtools. The non-synonymous (*Ka*) and synonymous (*Ks*) substitution rates for duplicated gene pairs were calculated using the *KaKs*_Calculator 2.0, and the *Ka*/*Ks* ratio was used to infer the selection pressure.

### 2.5. Gene Structure and Conserved Motif Analysis and Cis-Acting Regulatory Element Analysis in Promoters

Based on the genome annotation information, the exon, intron, and UTR details of the gene can be obtained, and the gene structure diagram was generated using the GSDS database (http://gsds.gao-lab.org/) [34]. To identify conserved motifs within the *RcKCS* proteins, the Multiple Em for Motif Elicitation (MEME) suite (version 5.5.2) was employed. The analysis was conducted with the following parameters: maximum number of motifs set to 10, and the optimum width of each motif between 6 and 50 amino acids. The resulting motifs were annotated by comparing them against the InterPro database. The 2000 bp genomic DNA sequences upstream of the transcription start site (TSS) of each *RcKCS* gene were extracted as putative promoter regions. These sequences were then submitted to the PlantCARE database for the identification of cis-acting regulatory elements. The results were filtered and categorized based on their known functions, and a schematic diagram of the distribution of these elements was generated using TBtools for visual representation.

### 2.6. Expression Analysis of RcKCS Family Members and Functional Analysis of RcKCS6 via Gene Cloning, Transient Overexpression, and Gene Silencing

Based on previously obtained transcriptomic data from rose (*Rosa × hybrida*) cultivar ‘Jumilia’ petals inoculated with *B. cinerea* at 0, 1, 2, 3, 4 days post-inoculation (dpi) (raw data deposited in the NCBI SRA database under accession number *PRJNA1209018*), the expression patterns of *RcKCS* family members were analyzed. To investigate the function of the *RcKCS6* gene, total RNA was extracted from rose petals and reverse-transcribed into cDNA [35]. Gene-specific primers were designed based on the *RcKCS6* gene sequence, and the target fragment was amplified by PCR and purified. For virus-induced gene silencing (VIGS), the amplified fragment was inserted into the TRV2 vector to construct the silencing plasmid [35]. For transient overexpression, the full-length coding sequence of *RcKCS6* was cloned into the pSuper1300 vector. After sequence verification, the recombinant constructs were transformed into *Agrobacterium tumefaciens* strain GV3101. The bacterial cultures were collected by centrifugation and resuspended in infiltration buffer to an OD_600_ of 1.6 [36].

Following the method described [35], rose petals were punched into circular disks with a diameter of 1.5 cm using a cork borer. The *Agrobacterium* suspension containing TRV1 was mixed with either TRV2 or TRV2-*RcKCS6* at a 1:1 ratio and infiltrated into the petal disks using a vacuum infiltration method. Petal disks were immersed in the infiltration solution under a vacuum pressure of 0.07 MPa for 5 min. After vacuum release, the disks were rinsed with deionized water and incubated at 8 °C in darkness for 3 days, followed by incubation in an artificial climate chamber at 22 °C/19 °C (day/night) with a 16 h/8 h photoperiod for another 3 days. At 6 days after TRV infiltration, *B. cinerea* was inoculated onto the petal disks. At least 16 petal disks were used per treatment, and the VIGS experiment was independently repeated at least three times. Lesion areas on the petals were measured and compared after inoculation. Primer sequences used for gene silencing and overexpression are listed (Table S2). *RhActin5* was used as the internal reference gene. qRT-PCR primers for RcKCS6 were designed using Primer 6.0 software. Quantitative real-time PCR was performed using the Hieff Universal Blue qPCR SYBR Green Master Mix (YEASEN) according to the manufacturer’s instructions. Relative gene expression levels of *RcKCS6* were calculated using the 2^−ΔΔCt^ method [37]. Primer sequences used for qRT-PCR analysis are provided (Table S3).

### 2.7. Statistical Analysis

All statistical analyses were performed using IBM SPSS Statistics 26. Data were expressed as mean ± standard deviation (SD) of at least three biological replicates. Differences between groups were evaluated using one-way analysis of variance (ANOVA), followed by Student–Newman–Keuls (SNK) post hoc test for multiple comparisons. A *p*-value < 0.05 was considered statistically significant. Error bars in all figures represent standard deviations.

## 3. Results

### 3.1. Genome-Wide Identification and Physicochemical Properties of RcKCS Genes

Through a comprehensive genome-wide analysis employing HMMER and BLASTp searches, followed by rigorous domain validation, we identified 18 non-redundant *KCS* genes in the *rose* genome (Table 1). These genes were systematically named *RcKCS1* to *RcKCS18* based on their order of appearance on the chromosomes (Table 1). The amino acid length ranged from 436 (*RcKCS12*) to 541 (*RcKCS18*), corresponding to molecular weights varying from approximately 48.49 kDa to 61.44 kDa. The theoretical isoelectric points (pI) exhibited a broad range from 8.08 (*RcKCS3*, slightly basic) to 9.75 (*RcKCS14*, strongly basic), suggesting variations in their charge characteristics under physiological conditions. The grand average of hydropathicity (GRAVY) scores were all close to zero, ranging from −0.18 (*RcKCS10*, slightly hydrophilic) to 0.08 (*RcKCS14*, slightly hydrophobic), indicating that these proteins are likely to be globular and soluble. Subcellular localization predictions using WolfPSORT suggested that the majority of *RcKCS* proteins are localized to the endoplasmic reticulum, which is consistent with their known role in VLCFA biosynthesis.

### 3.2. Phylogenetic Analysis and Classification of the RcKCS Family

**Figure 1:**
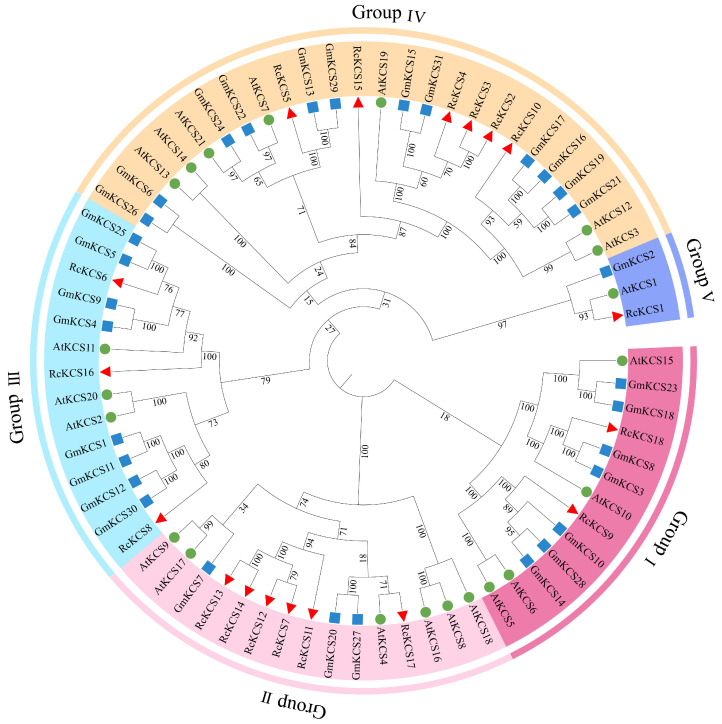
An unrooted neighbor-joining (NJ) phylogenetic tree based on the amino acid sequence alignment among *A. thaliana*, *G. max*, and *R. chinensis* KCS sequences with 1000 bootstraps. All the KCS members were divided into 5 groups and presented in different colors. The protein sequences of AtKCS, GmPeKCS, and RcKCS are represented by green, blue, and red color respectively.

To investigate the evolutionary relationships and classification of the RcKCS proteins, a phylogenetic tree was constructed using the Neighbor-Joining method, incorporating 70 KCS protein sequences from rose (18), Arabidopsis (21), and soybean (31). The phylogenetic analysis clearly segregated all KCS proteins into five distinct clades, with a bootstrap support threshold of ≥70% used to define the clades, which are designated as Group I to Group V (Figure 1). The distribution of RcKCS members across these groups was uneven. Group II and Group IV were the largest, each containing six RcKCS proteins. Group III contained three members, while Group I and Group V contained two and one member(s), respectively. Notably, RcKCS proteins within the same group often clustered more closely with soybean GmKCS proteins than with Arabidopsis AtKCS proteins, indicating a potentially closer evolutionary relationship between rose and soybean within the *KCS* family. This phylogenetic classification provides a framework for predicting potential functional redundancies or specializations among the RcKCS members.

### 3.3. Chromosomal Distribution and Gene Duplication Events

**Figure 2:**
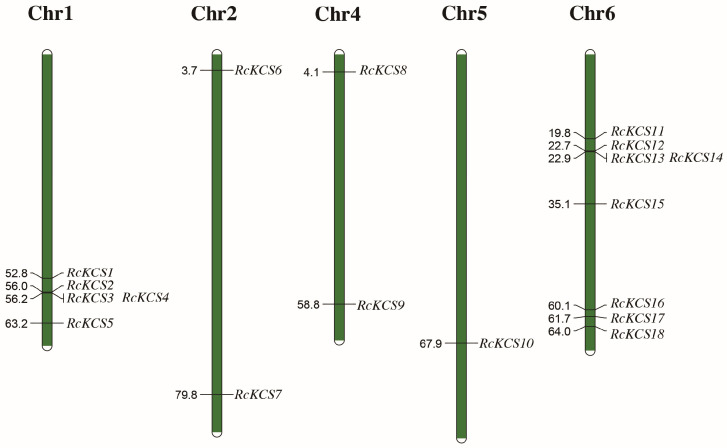
The 18 *RcKCS* genes are mapped onto five chromosomes of rose, with no distribution on the remaining chromosomes.

The 18 identified *KCS* genes were unevenly distributed across five of the seven rose chromosomes; notably, no *KCS* loci were detected on chromosomes 3 and 7 (Figure 2) [38]. Chromosome 6 harbored the largest number of *RcKCS* genes, with eight genes (*RcKCS11* to *RcKCS18*) clustered in a specific region. Chromosome 1 contained five genes (*RcKCS1* to *RcKCS5*), while chromosomes 2 and 4 each contained two genes (*RcKCS6–7* and *RcKCS8–9*, respectively). Chromosome 5 contained only one gene (*RcKCS10*). No *RcKCS* genes were located on chromosomes 3 and 7. This uneven distribution suggests that certain chromosomal regions may be hotspots for the evolution of this gene family.

**Figure 3:**
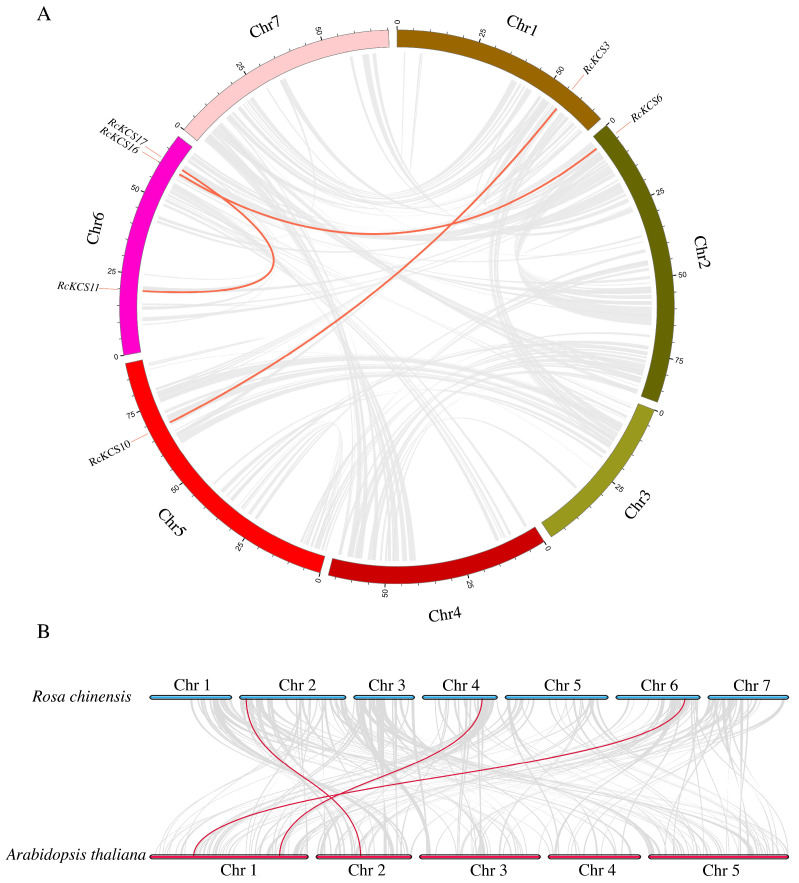
Synteny analysis of the *KCS* genes. (**A**) Synteny analysis of the *RcKCS* genes in *R. chinensis*. (**B**) Synteny analysis of the *KCS* genes between *R. chinensis* and *A. thaliana*. The red lines highlight the syntenic *KCS* gene pairs; the gray lines in the background represent collinear blocks that are orthologous to other plant genomes in roses.

To understand the potential mechanisms behind the expansion of the *RcKCS* family, we analyzed gene duplication events. MCScanX analysis identified three pairs of segmentally duplicated genes (*RcKCS3*/*RcKCS10*, *RcKCS6*/*RcKCS16*, and *RcKCS11*/*RcKCS17*) and one pair of tandemly duplicated genes (*RcKCS11*/*RcKCS17*, with *RcKCS11* and *RcKCS17* being part of the cluster on chromosome 6 and also identified as a segmental pair) (Figure 3A). The *Ka*/*Ks* ratios for all duplicated pairs were significantly less than 1, ranging from 0.04 to 0.08 indicating that the *RcKCS* gene family has undergone purifying selection, a form of natural selection that removes deleterious mutations and preserves functional sequences, thereby maintaining conserved functions (Table S3). Furthermore, synteny analysis between rose and Arabidopsis genomes revealed three orthologous *KCS* gene pairs (Figure 3B), suggesting that these genes have conserved functions dating back to their last common ancestor and may play fundamental roles in core lipid metabolic pathways.

### 3.4. Gene Structure and Conserved Motif Composition

**Figure 4:**
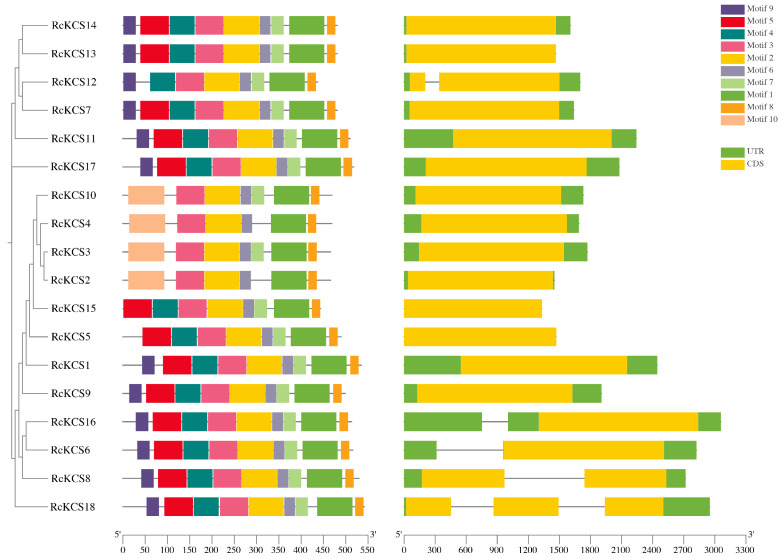
The unrooted phylogenetic tree, conserved motifs, and gene structure of *RcKCS* genes, where different colors represent different motifs.

The exon-intron structure of the *RcKCS* genes was analyzed to gain insights into their structural evolution (Figure 4). The analysis revealed a remarkable conservation, with the vast majority (15 out of 18) of *RcKCS* genes containing only a single exon, lacking introns entirely. Only three genes (*RcKCS12*, *RcKCS13*, and *RcKCS18*) contained introns, with *RcKCS18* having the highest number (3 exons). This predominant single-exon structure is a common feature of many *KCS* genes in plants and may facilitate rapid transcriptional responses to environmental cues.

Conserved motif prediction using MEME identified 10 distinct motifs (Motif 1–10) among the RcKCS proteins. Motif 1 and Motif 2 were universally present in all 18 members, and subsequent domain annotation confirmed that these two motifs correspond to the core FAE1_CUT1_RppA and ACP_syn_III_C domains, respectively. The composition and order of motifs were generally conserved within each phylogenetic group, reinforcing the reliability of our phylogenetic classification.

### 3.5. Analysis of Cis-Acting Regulatory Elements in Promoter Regions

**Figure 5:**
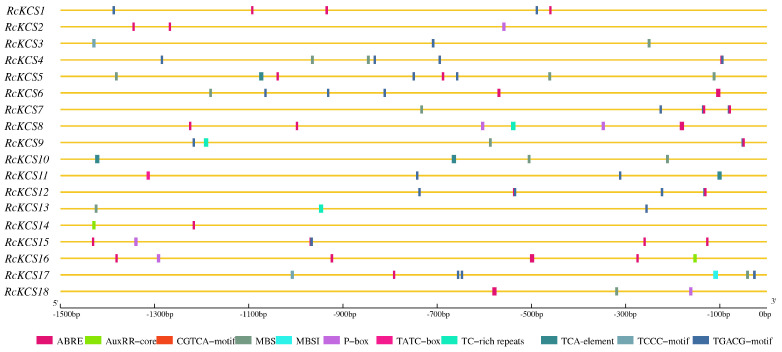
Predicted *cis*-elements in *RcKCSs* promoters. Different colors represent the different types of *cis*-elements.

To predict the potential regulatory mechanisms of *RcKCS* genes, we analyzed the cis-acting elements in their 2000 bp promoter regions (Figure 5). A plethora of elements associated with hormone responses, stress responses, and plant development were identified. Phytohormone-responsive elements were the most abundant, including abscisic acid-responsive elements (ABRE), auxin-responsive elements (AuxRR-core, TGA-element), MeJA-responsive elements (CGTCA-motif, TGACG-motif), gibberellin-responsive elements (P-box, GARE-motif), and salicylic acid-responsive elements (TCA-element). This suggests that the expression of *RcKCS* genes is likely finely tuned by complex hormonal networks, particularly those involved in defense signaling such as JA, SA, and ABA [39]. Furthermore, numerous stress-responsive elements were detected. These included MYB binding sites involved in drought-inducibility (MBS), low-temperature responsiveness (LTR), defense and stress responsiveness (TC-rich repeats), and fungal elicitor responsiveness (Box-W1). The widespread presence of these elements strongly implies that *RcKCS* genes play integral roles in roses’ adaptation to various abiotic and biotic stresses, including pathogen attack.

### 3.6. Expression Profiles of RcKCS Genes in Response to B. cinerea Infection

**Figure 6:**
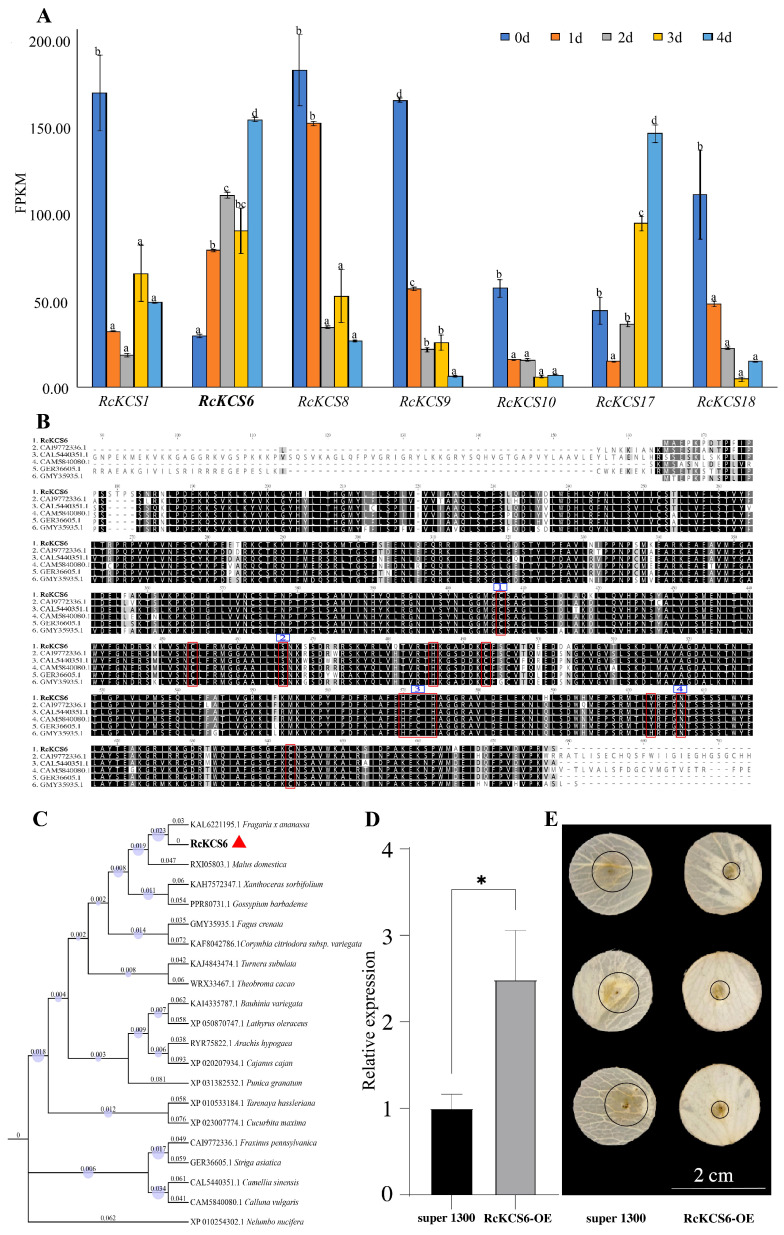
Expression analysis, sequence alignment, and functional characterization of *RcKCS6*. (**A**) Differential expression of *RcKCS* family members in rose cultivar ‘Jumilia’ petals at 0, 1, 2, 3, 4 days post-inoculation with *Botrytis cinerea*, showing significant upregulation of *RcKCS6*. Different letters indicate significant differences between groups (*p* < 0.05). (**B**) Multiple sequence alignment of *RcKCS6* with other KCS proteins, highlighting nine conserved catalytic sites critical for fatty acid elongation. (**C**) Phylogenetic tree showing the evolutionary relationship between *RcKCS6* and KCS proteins from *Fragaria × ananassa*. (**D**) Construction of transient overexpression for *RcKCS6* and its introduction into rose petals via *Agrobacterium*-mediated infiltration, * indicates a significant difference, *p* < 0.05. (**E**) Disease resistance assay showing that after *B. cinerea* infection, the lesion area on *RcKCS6*-overexpressing rose petals is significantly smaller compared to the control petals.

To explore the potential roles of *RcKCS* genes in the resistance of roses to gray mold, the expression patterns of *RcKCS* family members were first analyzed in petals of the rose cultivar ‘Jumilia’ at 0, 1, 2, 3, and 4 days after inoculation with *B. cinerea*. The detailed expression data are provided in Table S4 and were derived from previously generated transcriptome datasets. Among the *RcKCS* family members, seven genes exhibited significant differential expression during infection (Figure 6A). The expression levels of *RcKCS1*, *RcKCS8*, *RcKCS9*, *RcKCS10*, and *RcKCS18* showed a decreasing trend, whereas *RcKCS6* and *RcKCS17* were predominantly upregulated. Notably, the expression level of *RcKCS6* was markedly higher than that of *RcKCS17* at 1, 2, and 4 days post-inoculation (Figure 6A), suggesting that both *RcKCS6* and *RcKCS17* may be involved in rose resistance to gray mold, with *RcKCS6* showing a stronger association.

Based on these results, *RcKCS6* was selected for gene cloning and functional characterization. The full-length *RcKCS6* gene consisted of 1548 bp and encoded a protein of 516 amino acids. The complete nucleotide and deduced amino acid sequences are provided in Table S5. Multiple sequence alignment of *RcKCS6* with other KCS proteins, combined with previous studies on KCS proteins [13], revealed that nine conserved catalytic sites are essential for fatty acid elongation. Among these, four residues—cysteine (C), serine (S), histidine (H), and asparagine (N)—are critical for KCS enzymatic function (Figure 6B). Phylogenetic analysis further indicated that RcKCS6 is most closely related to the KCS protein from *Fragaria × ananassa* (Accession no. KAL6221195.1) (Figure 6C). To further investigate the role of *RcKCS6* in rose resistance to gray mold, transient overexpression and virus-induced gene silencing (VIGS) constructs of *RcKCS6* were successfully generated (Figure 6D) and introduced into rose petals via *Agrobacterium*-mediated infiltration. The disease resistance assays showed that after transient overexpression of *RcKCS6*, the lesion area on the petals was significantly smaller compared to the control, indicating that transient overexpression of *RcKCS6* can enhance resistance to *B. cinerea* to some extent (Figure 6E). These results provide preliminary evidence that overexpression of *RcKCS6* confers enhanced resistance to gray mold in rose.

## 4. Discussion

The *KCS* gene family plays a crucial role in the biosynthesis of very long-chain fatty acids (VLCFAs) by catalyzing the rate-limiting step of their elongation, determining both their biosynthetic rate and final carbon-chain length [13,40,41]. VLCFAs are essential for plant cells, serving as structural components or precursors for specialized metabolites [42]. They form key components of lipids, contributing to the formation of protective barriers that regulate plant development and stress responses [43,44]. In specialized tissues, VLCFAs are further used for the biosynthesis of cuticular waxes, pollen exine, and suberin, which are indispensable for root development. Cuticular waxes form a hydrophobic layer covering most aerial plant surfaces, preventing excessive water loss, regulating transpiration, limiting pathogen invasion, and enhancing tolerance to environmental stresses [8,40,45]. Additionally, VLCFAs are stored in the seeds of Brassicaceae species and jojoba as triacylglycerols and wax esters, representing a significant energetic investment by the maternal plant [42,43,44].

The *KCS* gene family has been identified in a wide range of plant species, and the number of family members varies considerably among taxa. Sixteen *KCS* genes have been reported in sweet orange (*Citrus sinensis*) [15], 18 in oil camellia (*Malania oleifera*) [13], 20 in *Xanthoceras sorbifolium* [16], 21 in *A. thaliana* [12], 22 in rice (*Oryza sativa*) [17], 25 in sorghum (*Sorghum bicolor*) [46], 28 in apple [47], 30 in peanut (*Arachis hypogaea*) [48], and as many as 58 in rapeseed (*Brassica napus*) [49]. In this study, we performed the first genome-wide identification and characterization of the *KCS* gene family in rose and identified 18 *RcKCS* genes. The number of *RcKCS* genes is identical to that in *M. oleifera* [13] and is comparable to those reported in *C. sinensis* [15], *X. sorbifolium* [16], and *Arabidopsis* (21 genes) [12], indicating that the *KCS* gene family in roses is of moderate size [12,16]. Phylogenetic analyses suggest that the *KCS* gene originated as a single-copy gene in the green plant lineage; subsequent gene duplication events generated five ancestral copies in land plants, forming five major monophyletic clades. Further duplication in angiosperms gave rise to 11 lineage-specific genes, which subsequently expanded to approximately 20–30 members in many flowering plant species [50].

Chromosomal localization analysis revealed that *RcKCS* genes are unevenly distributed across the rose genome, with a notable gene cluster on chromosome 6, indicating that gene family expansion is closely associated with gene duplication events. Consistent with this, our duplication analysis confirmed that segmental duplication was the primary driving force underlying the expansion of the *RcKCS* gene family, as also observed in other species such as passion fruit [51]. The Ka/Ks ratio is widely used to infer selection pressure, where Ka/Ks = 1 indicates neutral evolution, Ka/Ks > 1 indicates positive selection, and Ka/Ks < 1 indicates purifying selection [52]. All duplicated *RcKCS* gene pairs exhibited Ka/Ks ratios far below 1, indicating that these genes have evolved under purifying selection, reflecting the functional constraint and evolutionary importance of KCS enzymes in core metabolic processes [53]. Similarly, all seven duplicated gene pairs in the *A. thaliana* DUF4228 gene family also showed Ka/Ks values < 1, suggesting that this gene family has likewise experienced purifying selection following duplication events [54].

The conserved synteny between *RcKCS* genes and their *Arabidopsis* counterparts further suggests that these orthologs share ancient and evolutionarily conserved functions [16]. The high conservation of gene structure (predominantly single-exon genes) and motif composition within phylogenetic groups supports the hypothesis that members within the same clade may have redundant or similar biological functions. The ubiquitous presence of Motif 1 and Motif 2, which correspond to the catalytic domains, highlights their essential role in the condensation reaction during VLCFA elongation (Figure 4) [17,48]. In contrast, variation in other motifs among different clades may contribute to functional diversification, such as differences in substrate specificity or interactions with distinct protein partners.

Promoter analysis provided strong indirect evidence that *RcKCS* genes are involved in plant stress responses. A large number of hormone-responsive cis-elements (including ABRE, TGACG-motif, and TCA-element) and stress-related elements (such as MBS and TC-rich repeats) were identified, consistent with the known functions of *KCS* genes in other plant species [39,55,56,57,58]. For example, the presence of ABREs in the promoters of eight *RcKCS* genes suggests that they may be regulated by abscisic acid (ABA), a key hormone in abiotic stress responses. ABA has been shown to promote suberin accumulation in *Arabidopsis* roots [55], potato tubers [56], tomato fruits [57], and kiwifruit [58]. During wound-induced suberization in kiwifruit (*Actinidia deliciosa*), the *AchnKCS* promoter is activated through interaction with *AchnMYB70* and repressed by *AchnbZIP29*; ABA likely plays a critical role in the transcriptional activation of *AchnKCS* by upregulating *AchnMYB70* and downregulating *AchnbZIP29* [39]. Similarly, the presence of jasmonic acid (JA)- and salicylic acid (SA)-responsive elements suggests that *RcKCS* genes may participate in defense signaling pathways activated upon pathogen infection.

Roses are among the most economically important ornamental plants worldwide, accounting for more than one-third of the global cut flower market [2,59]. Gray mold, caused by the necrotrophic fungus *B. cinerea*, is one of the major postharvest diseases of roses and results in substantial economic losses [3,4]. When fungal pathogens do not enter plant tissues through natural openings or wounds but instead penetrate directly through the plant surface, they must overcome the cuticle and the epidermal cell wall, with the cuticle generally regarded as a passive physical barrier against pathogen invasion [60]. Statistical analyses have revealed a highly significant correlation between the relative impedance of cuticular wax and the resistance of grapevines to *B. cinerea* [61]. In tomato, abscisic acid deficiency leads to increased cuticle permeability, relatively higher levels of pectin methylesterification, and the release of distinct oligosaccharides upon *B. cinerea* infection, which together enhance resistance to gray mold [60].

Fungal pathogens initiate infection by secreting cutinases that degrade the plant cuticle and facilitate host penetration [62,63]. Because the cuticle consists mainly of cutin and waxes, whose wax fraction is largely composed of very long-chain fatty acids (VLCFAs) and their derivatives [11], its structural integrity depends on VLCFA biosynthesis. Reduced wax deposition and cuticle thickness have been shown to enhance *B. cinerea* penetration in blueberry [64], underscoring the importance of wax composition in resistance. As KCS catalyzes the rate-limiting step of VLCFA elongation, variation in KCS gene function may influence wax accumulation and cuticle architecture, thereby affecting plant susceptibility to pathogens. In this context, our genome-wide identification and characterization of the *RcKCS* gene family provide a molecular basis linking VLCFA biosynthesis to cuticle-associated defense in rose.

*KCS* genes encode the key and rate-limiting enzymes in the biosynthesis of very long-chain fatty acids (VLCFAs) [13,40,41]. To investigate the involvement of *RcKCS* genes in the interaction between rose and *B. cinerea*, we analyzed the differential expression patterns of *RcKCS* family members at different time points following *B. cinerea* infection in the rose cultivar ‘Jumilia’. Seven *RcKCS* genes were significantly differentially expressed during infection. Notably, the transcript level of *RcKCS6* increased continuously with the progression of infection, suggesting that *RcKCS6* may play a positive regulatory role during *B. cinerea* infection in rose (Figure 6A). Consistent with this hypothesis, previous studies have shown that overexpression of *Arabidopsis AtKCS2* enhances cuticular wax accumulation and drought tolerance [22], overexpression of apple *MdKCS2* has been observed to increase wax deposition [28], silencing of barley *HvKCS6* was found to alter wax composition [31], and overexpression of *HvKCS1* has been shown to increase leaf wax content and confer resistance to powdery mildew [32]. Furthermore, transient overexpression of *RcKCS6* in rose petals significantly enhanced resistance to gray mold, as evidenced by a marked reduction in lesion size. These results indicate that *RcKCS6* responds rapidly to pathogen challenge and contributes to resistance by promoting wax biosynthesis and reinforcing the cuticular barrier to restrict further pathogen invasion.

## 5. Conclusions

In this study, we performed a genome-wide identification and characterization of the *KCS* gene family in roses, identifying 18 *RcKCS* genes. These genes were analyzed for their phylogenetic relationships, chromosomal distribution, gene structure, duplication patterns, conserved motifs, cis-regulatory elements, and expression during *B. cinerea* infection. The *RcKCS* genes were mainly expanded through segmental duplication and under purifying selection, indicating functional conservation. Promoter analysis revealed stress- and hormone-responsive elements, suggesting a role in developmental regulation and stress responses. Notably, *RcKCS6* may play an important role in roses’ resistance to gray mold, offering a potential target for enhancing disease resistance. This study provides new insights into the *KCS* gene family’s evolution and its involvement in cuticular wax formation and disease resistance in rose. Further validation using CRISPR/Cas9 and functional profiling will be crucial for confirming these findings and understanding the regulatory networks involving *RcKCS* genes.

## Figures and Tables

**Table 1:** Summary of the KCS gene family identified in rose (*Rosa × hybrida* Hort.).

Name	Gene ID	Chromosome No.	Location (Start–End)	MW (Da)	Protein Length (aa)	Amino Acid No.	PI	GRAVY Value
*LOC112182122*	*RcKCS1*	NC_037088.1	52,753,860–52,756,304	59,930.6	534	534	9.45	−0.14
*LOC112180806*	*RcKCS2*	NC_037088.1	55,993,322–55,994,773	52,529.8	465	465	8.41	0.065
*LOC112186018*	*RcKCS3*	NC_037088.1	56,208,369–56,210,139	52,579.6	465	465	8.08	0.011
*LOC112170275*	*RcKCS4*	NC_037088.1	56,216,138–56,217,826	52,833.1	468	468	9.09	−0.017
*LOC112171831*	*RcKCS5*	NC_037088.1	63,193,360–63,194,829	54,685.1	489	489	9.27	−0.095
*LOC112186661*	*RcKCS6*	NC_037089.1	3,686,800–3,689,622	58,046.9	515	515	9.38	−0.093
*LOC112189629*	*RcKCS7*	NC_037089.1	79,819,236–79,820,876	53,337.8	480	480	9.68	0.074
*LOC112198004*	*RcKCS8*	NC_037091.1	4,120,956–4,123,674	59,656	529	529	9.55	−0.056
*LOC112198227*	*RcKCS9*	NC_037091.1	58,799,843–58,801,749	56,117	498	498	8.98	0.025
*LOC112201921*	*RcKCS10*	NC_037092.1	67,914,310–67,916,040	52,946.8	468	468	8.83	−0.177
*LOC112174521*	*RcKCS11*	NC_037093.1	19,749,321–19,751,564	56,896.6	509	509	9.28	−0.023
*LOC112171065*	*RcKCS12*	NC_037093.1	22,712,332–22,714,032	48,492.1	436	436	9.63	0.033
*LOC112174779*	*RcKCS13*	NC_037093.1	22,857,559–22,859,025	53,746.1	480	480	9.56	0.06
*LOC112168776*	*RcKCS14*	NC_037093.1	22,939,580–22,941,186	53,887.5	480	480	9.75	0.076
*LOC112171233*	*RcKCS15*	NC_037093.1	35,105,474–35,106,805	49,510.6	443	443	9.34	−0.013
*LOC112169542*	*RcKCS16*	NC_037093.1	60,052,915–60,055,973	57,817.9	512	512	9.56	−0.048
*LOC112169592*	*RcKCS17*	NC_037093.1	61,668,213–61,670,291	57,693.3	517	517	9.54	−0.058
*LOC112171863*	*RcKCS18*	NC_037093.1	63,957,632–63,960,583	61,440.1	541	541	9.1	−0.171

MW: molecular weight (kDa); PI: theoretical isoelectric point; GRAVY: Grand Average of Hydropathy.

## Data Availability

All data generated or analyzed during this study are included in this published article and its Supplementary Information Files.

## Supplementary Materials

The following supporting information can be downloaded at https://www.mdpi.com/article/10.3390/genes17030286/s1: Table S1: Protein ID and sequence information; Table S2: Primer sequences for *RcKCS6* gene analysis; Table S3: The Ka/Ks ratios of the duplicated *RcKCS* gene pairs; Table S4: Detailed expression data of *RcKCS* family members in rose cultivar ‘Jumilia’ petals at 0, 1, 2, 3, 4 days post-inoculation with *Botrytis cinerea* (derived from transcriptome datasets); Table S5: Complete nucleotide and deduced amino acid sequences of the *RcKCS6* gene.
